# Dietary Pyridoxine Requirements of Coho Salmon (*Oncorhynchus kisutch*) Post-Smolts

**DOI:** 10.1155/2024/3862563

**Published:** 2024-10-08

**Authors:** Hairui Yu, Xinyue Zhang, Ziyi Yuan, Leyong Yu, Youzhi Zhao, Lingyao Li

**Affiliations:** ^1^Key Laboratory of Biochemistry and Molecular Biology in Universities of Shandong (Weifang University), Weifang Key Laboratory of Coho Salmon Culturing Facility Engineering, Institute of Modern Facility Fisheries, College of Biology and Oceanography, Weifang University, Weifang 261061, China; ^2^Weifang Centre for the Promotion of Scientific and Technological Innovation, Weifang 261000, China; ^3^Shandong Collaborative Innovation Center of Coho Salmon Health Culture Engineering Technology, Shandong Conqueren Marine Technology Co. Ltd., Weifang 261108, China; ^4^Conqueren Leading Fresh Science and Technology Inc. Ltd., Weifang 261205, China

**Keywords:** growth performance, *Oncorhynchus kisutch* post-smolts, physiological metabolism, pyridoxine

## Abstract

A 10-week feeding trial was conducted to investigate the dietary pyridoxine requirements of coho salmon (*Oncorhynchus kisutch*) post-smolts with an initial mean body weight of 180.22 ± 0.41 g. Seven diets were prepared with gradient pyridoxine levels of 0.32, 1.25, 2.56, 4.08, 8.24, 16.02, and 32.32 mg/kg, respectively, and each diet was assigned to three replication groups of 10 fish. The results revealed that coho salmon fed the diet with pyridoxine supplementation gained more final body weight (FBW), specific growth rate (SGR), and better feed conversion ratio (FCR). FBW and SGR of the fish fed the diet with 8.24 mg/kg pyridoxine were significantly higher than those of the other groups (*P* < 0.05). An inverse trend was observed for FCR, which was the lowest in fish fed the diet with 8.24 mg/kg pyridoxine. The gradient pyridoxine levels did not yield any statistically (*P* > 0.05) significant impact on the whole-body composition including moisture, ash, crude lipid, and crude protein. The hepatic pyridoxine concentration, aspartate aminotransferase (AST), and alanine aminotransferase (ALT) activities increased significantly with dietary pyridoxine levels increasing from 0.32 to 4.08 mg/kg (*P* < 0.05) and plateaued after that (*P* > 0.05). The coho salmon fed the diet with 8.24 mg/kg pyridoxine achieved the maximum superoxide dismutase and catalase, as well as the minimum total cholesterol, triglyceride, and malondialdehyde. Broken line analysis of SGR, FCR, AST, and ALT activities reflected the optimal dietary pyridoxine requirements for coho salmon post-smolts from 3.92 to 7.08 mg/kg diet.

## 1. Introduction

Pyridoxine (vitamin B_6_), a water-soluble vitamin, is an indispensable nutrient for animals [1]. The enzymatically active form of pyridoxine in organisms, known as pyridoxal-5-phosphate (PLP), is a coenzyme in the enzymatic system of protein metabolism, antioxidant activity, antihyperlipidemic, and hepatoprotective properties [2–4]. Pyridoxine would affect the body composition and nutritional value by regulating these systems. In rats, pyridoxine reduced triglyceride accumulation and lower hepatic fat storage [4]. The lack of pyridoxine in fish could lead to poor appetite, suppressed immune function, and even histopathological changes [5, 6]. Relevant studies have shown that these symptoms were related to the inhibition of transaminase activity and cell antioxidant defense system [7]. The pyridoxine required for farmed fish is mainly provided by adding to their diet [8]. Therefore, determining the optimal level of pyridoxine in diets is conducive to the growth of farmed fish.

Pyridoxine brings many health benefits to fish. Based on the previous studies, the pyridoxine demands were different for fish in different species and different growth stages, for example, the optimal dietary pyridoxine requirements of trout, salmon, carp, and sea bream were 10–15, 15–20, 5–10, and 5–6 mg/kg diet, respectively [9]. The optimal dietary intakes of pyridoxine were also determined in juvenile golden pompano (*Trachinotus ovatus*) at 8.84–9.28 mg/kg [10], and juvenile yellow catfish (*Pelteobagrus fulvidraco*) at 6.70–7.42 mg/kg [11]. Except for species and growth stages of fish, other factors including dietary protein levels, water temperature, and skin diseases have also been reported to influence the pyridoxine demand for fish [12–14].

Coho salmon (*Oncorhynchus kisutch*) is a temperate fish species that mainly lives in the north Pacific Ocean. It has been one of the most valuable Pacific salmon in commercial and sport fisheries. In recent years, coho salmon has become increasingly popular with consumers around the world due to its high-quality protein and unsaturated fatty acids. Although the salmon farming industry is booming, studies on coho salmon nutrition are still incomplete of which pyridoxine requirement has not been reported. In order to improve its culture, there is an urgent need to develop nutritionally balanced diets based on pyridoxine requirement. The objective of the present study was to investigate the optimal dietary pyridoxine level of coho salmon in terms of growth performance, body composition, activities of liver enzyme and serum antioxidant enzyme, which has a certain practical guiding for the production of the coho salmon feed.

## 2. Materials and Methods

### 2.1. Diets Preparation

The formulae of the experimental diets, as well as proximate composition, were presented in Table 1. The purified ingredients were supplied by Shandong Conqueren Marine Technology Co., Ltd. (Weifang, China). The diet had roughly 9% crude fat and 42% crude protein. By adding varying amounts of pyridoxine hydrochloride (98% purity, Heowns Biochem Technologies Co., Ltd., Tianjin, China) at 0, 1.02, 2.08, 4.33, 9.13, 18.04, and 35.06 mg/kg in the diet, seven experimental diets were made with analyzed the dietary pyridoxine concentration (0.32, 1.25, 2.56, 4.08, 8.24,16.02, and 32.32 mg/kg). All the diets were stored at −20°C until further use.

The dietary pyridoxine concentration was evaluated according to GB/T 14702-2018. The samples were finely ground and extracted in 0.39% NaH_2_PO_4_ with an ultrasonic bath for 20 min. The extraction solution was filtered by microfiltration membrane (0.45 μm) and analyzed using Agilent HP1100 HPLC (Agilent Technologies, Waldbronn, Germany) with an ultraviolet detector at a detection wavelength of 290 nm. The column was an C18 column (250 mm × 4.6 mm, 5 μm; Agilent, Palo Alto, CA). The mobile phase consisted of a 0.39% aqueous solution of NaH_2_PO_4_ (solution A) and methanol (solution B) with following gradient program: solution A: 0–3 min, 99%–88%; 3–6.5 min, 88%–70%.

### 2.2. Rearing Trial

The coho salmon post-smolts were provided by Conqueren Leading Fresh Marine Science and Technology Inc., Ltd. (Weifang, China), and cultured in the research center of the same incorporation. In an earthen pond, 21 floating cages (1.0 m × 1.0 m × 1.0 m, L × W × H) were randomly assigned to 210 post-smolts with an initial mean body weight of 180.22 ± 0.41 g (10 fish/cage). Each experimental diet was randomly distributed to the three cages. The water supplied in this experiment was filtered underground cold water. Temperature (14 ± 1°C), dissolved oxygen levels (8.0 ± 0.2 ppm), and pH (7.0 ± 0.1) were identical across the cages. The coho salmon were fed three times daily (7:00, 12:00, 17:00) to apparent satiation during the 10-week feeding period. The coho salmon were kept under a natural day-to-night cycle.

### 2.3. Sampling Procedures

The coho salmon were starved for 24 h to sample following the end of the 10-week rearing trial. The fish in each cage were recorded the number and weighed. Three fish were randomly sampled from each cage, anesthetized by tricaine methane sulfonate (25 mg/L) and were rapidly dissected and then removed muscle, stored at −80°C to analyze the muscle proximate composition. The muscle samples were taken from the same position of each fish. Five other fish were randomly selected from each cage and measured their weight and body length after anesthetized by tricaine methane sulfonate (25 mg/L), and their blood was collected via caudal venipuncture with a disposable medical syringe. The serum was separated by centrifugation (3500 g, 15 min, 4°C) of the blood samples after storage at room temperature for 2 h. Once the blood was drawn, the five salmon underwent dissection, during which the liver and viscera were removed for weighing rapidly. The liver and serum samples were kept at −80°C until the antioxidative enzyme activities and biochemical parameters, transaminase activity, and pyridoxine concentrations were determined, respectively.

### 2.4. Analytical Methods

#### 2.4.1. Growth Performance

The calculations formulae were as follows

  $$\begin{array}{c} \text{Survival rate }\left(\text{SR},\%\right)=\frac{\text{Final amount of fish}}{\text{Initial amount of fish}}\times 100. \end{array}$$

  $$\begin{array}{c} \text{Specific growth rate }\left(\text{SGR},\%/\text{day}\right)=\frac{\left[\ln\left(\text{final body weight},g\right)-\ln\left(\text{initial body weight},g\right)\right]}{\text{Day}}\times 100. \end{array}$$

  $$\begin{array}{c} \text{Condition factor }\left(\text{CF},g/{\text{cm}}^{3}\right)=\frac{\text{Body weight},g}{{\left(\text{Body length},\text{cm}\right)}^{3}}\times 100. \end{array}$$

  $$\begin{array}{c} \text{Hepatosomatic index }\left(\text{HSI},\text{\% }\right)=\frac{\text{Liver weight},g}{\text{Body weight},g}\times 100. \end{array}$$

  $$\begin{array}{c} \text{Viscerosomatic index }\left(\text{VSI},\text{\% }\right)=\frac{\text{Viscera weight},g}{\text{Body weight},g}\times 100. \end{array}$$

  $$\begin{array}{c} \text{Feed conversion ratio }\left(\text{FCR}\right)=\frac{\text{Total feed intake},g}{\text{Final body weight},g-\text{Initial body weight},g}. \end{array}$$

#### 2.4.2. Proximate Composition Analysis

The moisture was assessed by drying the samples to a constant weight at 105°C. The ash was evaluated by incinerating the samples in a muffle furnace at 550°C for 4 h. The crude protein of the diets and whole-body composition was determined by the Kjeldhal apparatus with a conversion factor of 6.25. The crude lipid was extracted using petroleum ether by soxhlet apparatus [15].

#### 2.4.3. Enzymes and Biochemical Analysis

Total cholesterol (TC), triglyceride (TG) contents, superoxide dismutase (SOD), catalase (CAT) activities, and malondialdehyde (MDA) content in the serum were assayed with commercially available kits (Jianchenng Bioengineering Inst., Nanjing, China) using a microplate reader (Spark10M, Tecan, Männedorf, Switzerland).

TC and TG were assessed by COD-PAP method and GPO-PAP method, respectively. Both operation methods were adding 2.5 μL of serum and 250 μL of enzyme working solution into each well of the 96-well microplate and incubating at 37°C for 10 min, then measuring the absorbance value with a microplate reader at 500 nm detection wavelength.

The SOD and CAT activities were assessed by the hydroxylamine method and the ammonium molybdate method [16, 17], respectively. The SOD reaction was started by mixing 20 μL of serum, 20 μL of SOD working solution, and 200 μL of substrate working solution in a 96-well microplate. The reaction system was incubated for 20 min at 37°C. The absorbance measured at 450 nm detection wavelength. The CAT reaction system was incubated for 1 min at 37°C and terminated the reaction by adding ammonium molybdate, the absorbance was monitored at 405 nm. The MDA content was assessed by the thiobarbituric acid method, the reaction condition was 95°C water bath for 40 min, and then centrifuged at 4000 × *g* for 10 min after cooling. The absorbance of the supernatant was at 532 nm detection wavelength [18].

Each hepatic sample was placed in a stoppered test tube with adding nine volumes (w/v) of sterilized physiological saline, homogenized under ice-cold water bath conditions for 2 min followed by centrifugation (6000 rpm, 20 min, 4°C). The supernatant was collected to assess alanine aminotransferase (ALT) and aspartate aminotransferase (AST) activities and pyridoxine concentration. The ALT and AST activities were measured at 510 nm detection wavelength as described by Reitman and Frankel [19]. The hepatic pyridoxine concentration was analyzed by the same method as the dietary pyridoxine concentration.

### 2.5. Statistical Analyses

The data were displayed as mean with standard errors. The SPSS 25.0 software was applied for all statistical analyses. The data were analyzed by one-way analysis of variance (ANOVA), after verifying normality and homogeneity of variance, ascertaining the significant differences among the various treatments by Duncan's test. Statistical significance was at a 5% level. Based on SGR, FCR, ALT, and AST activities, the broken line regression analyses were used to estimate the optimal dietary pyridoxine requirements.

## 3. Results

### 3.1. Growth Performance

**Figure 1:**
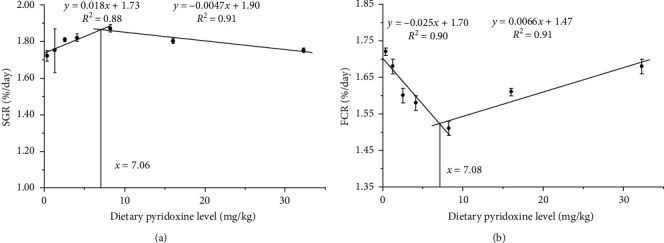
The effect of dietary pyridoxine on (A) specific growth rate (SGR) and (B) feed conversion ratio of coho salmon. Values in the figure represent means ± SE (*n* = 3). Requirements derived with the broken line method for SGR and FCR are 7.06 and 7.08 mg/kg diet, respectively.

The survival rate (SR) of the coho salmon fed the diet containing graded pyridoxine contents ranged from 96.67% to 100%, which had no (*P* > 0.05) significant difference among the groups (Table 2). The post-smolts fed the pyridoxine supplemented diet exhibited increased final body weight (FBW), SGR, and decreased FCR. The FBW and SGR of the coho salmon fed the diet with a pyridoxine content of 8.24 mg/kg were significantly higher than those of the other groups (*P* < 0.05). The FCR showed an opposite trend with the SGR and FBW, which was the best in the group of 8.24 mg/kg pyridoxine diet. The contents of CF, HSI, and VSI had no (*P* > 0.05) significant differences among the groups. Based on broken line regression analyses of SGR and FCR, the optimal dietary pyridoxine requirements were 7.06 mg/kg and 7.08 mg/kg, respectively (Figure 1).

### 3.2. Muscle Proximate Composition

The muscle proximate composition of coho salmon fed the diets with varying pyridoxine levels were presented in Table 3. The contents of moisture, crude protein, crude lipid, and ash ranged between 70.05%–70.84%, 13.09%–13.27%, 4.82%–4.85%, and 3.21%–3.72%, respectively. No (*P* > 0.05) differences were found for moisture, crude lipid, and ash among all the treatment groups.

### 3.3. Pyridoxine Concentration and ASTand ALT Activities in Liver

**Figure 2:**
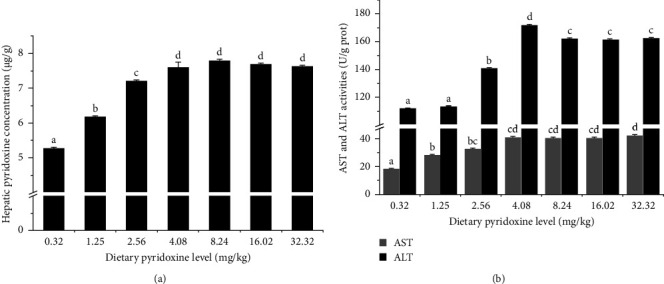
The pyridoxine concentration and AST and ALT activities in the liver of coho salmon after feeding the experimental diets for 10 weeks. (A) Hepatic pyridoxine concentration; (B) AST and ALT activities. Values in the figure represent means ± SE (*n* = 3). Superscript bearing different letters (a, b, c, d) indicates (*P*  < 0.05) significant differences for the same enzyme.

**Figure 3:**
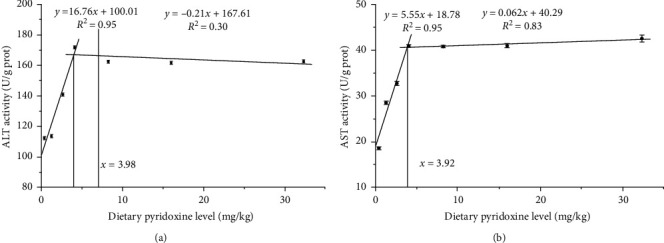
The effect of dietary pyridoxine on hepatic ALT activity (A) and AST activity (B) of coho salmon. Values in the figure represent means ± SE (*n* = 3). Requirements derived with the broken line method for ALT activity and AST activity are 3.98 and 3.92 mg/kg diet, respectively.

The hepatic pyridoxine concentration and AST and ALT activities are shown in Figure 1. The hepatic pyridoxine concentration increased with the increase of dietary pyridoxine level, and reached a peak of 7.78 µg/g when the pyridoxine level was 4.08 mg/kg, and subsequently leveling off at higher pyridoxine levels (Figure 2A). The trend of ALT activity was similar to hepatic pyridoxine concentration, which was reaching a peak of 162.05 U/g prot while the dietary pyridoxine level was 4.08 mg/kg. The findings revealed that the fish fed the diet containing 4.08 mg/kg pyridoxine exhibited significantly higher AST activity than the groups that consumed the diet containing ≤1.25 mg/kg pyridoxine (*P* < 0.05). However, there was no (*P* > 0.05) significant difference in AST activity observed among the groups that consumed diets containing 4.08–32.32 mg/kg pyridoxine (Figure 2B). The optimal pyridoxine requirements were reckoned using broken line regression analyses to be 3.92 and 3.98 mg/kg based on AST and ALT activities (Figure 3), respectively.

### 3.4. CAT and SOD Activities and MDA Content in Serum

The CAT, SOD activities, and MDA content in the serum were presented in Table 4. The increasing dietary levels of pyridoxine boosted the SOD and CAT activities (*P* < 0.05). The CAT and SOD activities increased with rising dietary pyridoxine levels up to 8.24 mg/kg and subsequently decreased with the pyridoxine levels further increasing (*P* < 0.05), as well as the MDA content significantly reduced when pyridoxine level up to 8.24 mg/kg (*P* < 0.05).

### 3.5. TC and TG Level in Serum

**Figure 4:**
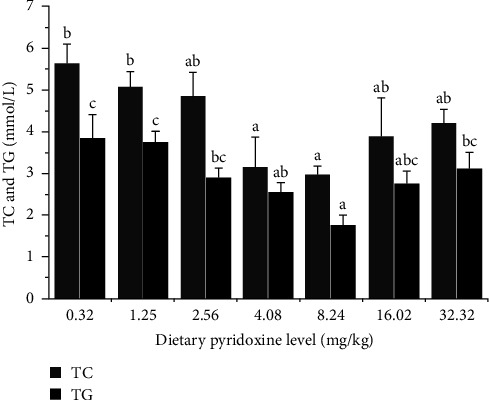
Total cholesterol and triglyceride in serum of coho salmon after feeding the experimental diets for 10 weeks. TC, total cholesterol; TG, triglyceride.

The contents of TC and TG in serum were provided in Figure 4, which exhibited a (*P* < 0.05) significant decrease when the dietary pyridoxine level increased within the range of 0.32–8.24 mg/kg. Subsequently, there was a modest increase in TC and TG with increasing dietary pyridoxine levels within the range of 8.24–32.32 mg/kg (*P* > 0.05).

## 4. Discussion

The levels of dietary pyridoxine influenced the growth performance of the coho salmon in this study. When increasing the dietary pyridoxine content up to 8.24 mg/kg, the growth rate and feed conversion efficiency were greatly enhanced. The optimal dietary pyridoxine content based on SGR and FCR was estimated to be 7.06–7.08 mg/kg, which is close to that of yellow catfish (6.90–6.93 mg/kg) [11], and Indian major carp (*Cirrhinus mrigala*; 5.63–6.05 mg/kg) [20]. However, it was higher than the requirement of 3 mg/kg for red hybrid tilapia (*Oreochromis mossambicus* × *O. niloticus*) found by Lim et al. [21], lower than that of 23 mg/kg for haliotis (*Discus hannai*) reported by Mai et al. [22]. The variation of pyridoxine requirement in the various species of fish might be owing to the difference in growth stage, protein level, analytical method, and assessment criteria [9, 13].

Pyridoxine deficiency affected the normal metabolic processes of protein and amino acids, resulting in reduced efficiency of protein absorption and utilization in the body and eventually leading to anorexia, growth retardation, anemia, and various other symptoms [9]. However, in the present study, the coho salmon without pyridoxine supplementation grew slowly, but none above symptoms occurred, which may be due to the short feeding period. Nile tilapia (*O. niloticus*) with pyridoxine deficiency did not show any of these abnormalities in a 13-week experiment [23], which is consistent with the present result. However, symptoms including tetany, nervous disorder, and blue-green color were reported in channel catfish (*Ictalurus punctatus*) with pyridoxine deficiency [24], and the symptom of erratic swimming was also observed in pyridoxine deficient grouper (*Epinephelus coioides*) [7].

In this study, there was no difference in the proximate composition of the muscle among the groups (*P* > 0.05). Similarly, a study of rainbow trout (*Oncorhynchus mykiss*) showed no (*P* > 0.05) significant differences for proximate composition both in fillet and whole body among different dietary pyridoxine levels during a 60-day feeding trial [25]. Additionally, Albrektsen, Waagbø, and Sandnes [26] discovered that a high-level of dietary pyridoxine did not have any impact on the moisture and protein composition of Atlantic salmon (*Salmo salar*) throughout a 20-week feeding trial, but lowered the lipid composition (*P* < 0.05). Cui et al. [27] found that the muscle composition was not affected by the level of dietary pyridoxine. As a water-soluble vitamin, pyridoxine has a relatively fast metabolic rate in the body, and its level is low in muscles, which might be the reason why it is difficult to affect muscle composition.

During the digestion process, pyridoxine is absorbed in the intestine and proceeds to the liver, where it is phosphorylated to PLP by pyridoxal kinase [28, 29]. The liver is the primary location for pyridoxine metabolism and activation in fish so hepatic pyridoxine concentration is commonly utilized to assess pyridoxine status [8, 25, 30]. The result of the present study suggested that the dietary pyridoxine level of 4.08 mg/kg might be sufficient to provide the nutrition for the liver metabolic function of coho salmon post-smolts. Comparable findings were observed in golden pompano [10] and grass shrimp (*Penaeus monodon*) [31].

The liver serves as a pivotal organ in charge of the metabolism of amino acids. Two transaminases, AST and ALT, are predominantly in the liver and play a crucial role in reflecting the metabolic and conversion function of amino acids [32]. PLP, the phosphate form of pyridoxine, is an enzyme cofactor required for methionine catabolism and cysteine synthesis in organisms [2, 33]. Pyridoxine and aminotransferase jointly participate in the catabolism of amino acids, so AST and ALT levels have been employed as indexes to reflect pyridoxine utilization in some fish [5]. According to the present study, ALT and AST activities increased with increasing dietary pyridoxine content, and the trend was comparable to that of hepatic pyridoxine concentration. A similar result was observed by Hemre et al. [34] who found that the muscle AST activity was correlated to muscle pyridoxine concentration in Atlantic salmon (*S. salar*). Our finding confirmed the role of pyridoxine in regulating the metabolism of amino acids in coho salmon.

The optimal dietary pyridoxine requirements based on AST and ALT activities for coho salmon were 3.92 and 3.98 mg/kg in this study, respectively, which were close to that of Indian catfish (*Heteropneustes fossilis*) at 3.21 mg/kg [35] and lower than golden pompano at 8.84–8.88 mg/kg [9]. In the present study, the optimal dietary pyridoxine contents based on ALT and AST were lower than those based on SGR and FCR. This discrepancy could be attributed to the asynchronous relationship between hepatic pyridoxine accumulation and growth. Several studies have demonstrated comparable findings: Cui et al. [27] detected that the optimal dietary pyridoxine based on WG and SGR (110.08–110.39 mg/kg) in juvenile Pacific white shrimp (*Litopenaeus vannamei*) was significantly lower than that based on the hepatopancreas pyridoxine concentration (167.5 mg/kg). Zehra and Khan [20] reported that the optimal dietary pyridoxine content of fingerling Indian major carp based on SGR and hepatic pyridoxine concentration were 5.63 and 8.61 mg/kg, respectively. Khan and Khan [36] reported that the optimal dietary pyridoxine content of fingerling major carp (*Catla catla*) based on FCR and hepatic pyridoxine concentration were 3.20 and 6.87 mg/kg, respectively. Growth performance and transaminase activity are two principal evaluation criteria for determining the optimal dietary pyridoxine requirement [5, 9]. Enzymatic activity, along with several other parameters such as protein content and fat deposition, influence fish growth performance, leading to growth, and enzyme activity out of synchronization [37, 38]. Consequently, these factors contribute to variations from different evaluation criteria.

In the studies of the Salmonidae family, the pyridoxine requirement varies from study to study [39]. The recommended dietary pyridoxine contents for salmon by NRC [5], and Halver and Hardy [9] were 4–7 mg/kg and 15–20 mg/kg, respectively. Albrektsen, Waagbø, and Sandnes [26] found the pyridoxine requirement for Atlantic salmon was 2–8 mg/kg, which is similar to the research result by Lall and Weerakoon [40] at 5 mg/kg, lower than that by Hemre et al. [34] at 10–16 mg/kg. The optimal dietary pyridoxine requirement for coho salmon post-smolts in the present study was 3.92–7.08 mg/kg. The differences between studies stemmed from complex changes in experimental conditions, including the composition of dietary ingredients (plant-based or fishmeal-based), the growth stage of salmon and the determination method of dietary pyridoxine.

The serum biochemical parameters of fish are usually regarded as health condition indicators, which are beneficial for determining the health state of fish in response to nutritional supplementation [41, 42]. In eukaryotic organisms, the antioxidant metalloenzymes CAT and SOD can remove excess free radicals from the body, prevent radical damage to the heart, kidney, and nervous system, and boost human immunity [43, 44]. In the present study, pyridoxine supplementation could improve SOD and CAT activities in the serum of coho salmon, corresponding to the finding of Pacific white shrimp supplied with dietary pyridoxine [27]. Furthermore, Zheng et al. [12] found that pyridoxine supplementation increased CAT and SOD mRNA levels in grass carp (*Ctenopharyngodon idella*) with pathogenic bacteria infection.

MDA is the end-product from the peroxidation reaction of free radicals on lipids. MDA content could reflect the extent of cellular damage and tissue peroxidation. In the present study, the decrease of MDA content occurred in the fish fed the diet with 2.56–8.24 mg/kg pyridoxine, which supported researches on several other fish species including grass carp [45] and yellow catfish [11]. These findings suggested that pyridoxine could improve serum antioxidative activities of aquatic animals.

In addition to amino acid metabolism and antioxidation, pyridoxine also participates in several stages of fat metabolism involving transport, synthesis, and deposition [4, 46]. Triglyceride and cholesterol are primary constituents of blood lipids and are predominantly transported in serum. Consequently, the serum TG and TC levels could be used to assess the effect of pyridoxine on lipid metabolism. A rising level of TG is linked to a higher risk of cardiovascular disease [47]. In the present study, pyridoxine supplementation significantly reduced the serum TG and TC levels of coho salmon, consistent with studies of grass carp [48], and mandarin fish (*Siniperca chuatsi*) [49]. Our result confirmed that dietary pyridoxine has the potential to modulate lipid levels in coho salmon, which could prevent high cholesterol and the development of fatty liver.

## 5. Conclusions

In summary, this study demonstrated that pyridoxine supplementation in diet benefited the antioxidant capabilities, blood lipid levels, growth and feed conversion of coho salmon post-smolts. Based on the SGR, FCR, AST, and ALT activities, the optimal dietary pyridoxine requirements were 7.06, 7.08, 3.92, and 3.98 mg/kg using broken line regression analyses, respectively. Based on the current research, the most suitable range of pyridoxine addition in feed for coho salmon post-smolts was 4–7 mg/kg.

## Figures and Tables

**Table 1:** Ingredients and proximate composition of the experimental diets for coho salmon (% in dry matter).

	Dietary pyridoxine levels (mg/kg)						
	0.32	1.25	2.56	4.08	8.24	16.02	32.32
Ingredient (%)							
Casein	38.00	38.00	38.00	38.00	38.00	38.00	38.00
Gelatin	12.00	12.00	12.00	12.00	12.00	12.00	12.00
Corn oil	6.00	6.00	6.00	6.00	6.00	6.00	6.00
Fish oil	3.00	3.00	3.00	3.00	3.00	3.00	3.00
Dextrin	28.00	28.00	28.00	28.00	28.00	28.00	28.00
* α*-cellulose	8.00	8.00	8.00	8.00	8.00	8.00	8.00
Mineral premix^1^	2.50	2.50	2.50	2.50	2.50	2.50	2.50
Ca(H_2_PO_4_)_2_	1.50	1.50	1.50	1.50	1.50	1.50	1.50
Pyridoxine-free vitamin mixture^2^	1.00	1.00	1.00	1.00	1.00	1.00	1.00
Pyridoxine HCl (mg/kg)	0.00	1.02	2.08	4.33	9.31	18.04	35.06
Proximate analysis (%)							
Crude protein	42.42	42.22	42.73	42.31	42.52	42.66	42.47
Crude lipid	9.42	9.44	9.52	9.39	9.24	9.47	9.51
Crude ash	11.96	11.98	12.03	12.11	11.95	11.92	12.06
Pyridoxine (mg/kg)	0.32	1.25	2.56	4.08	8.24	16.02	32.32

^1^Mineral premix supplied the following (mg kg^−1^ mineral premix): AlK(SO_4_)_2_·12H_2_O, 123.7; CaCl_2_, 17,880.0; CuSO_4_ · 5H_2_O, 32.0; CoCl_2_ · 6H_2_O, 49.0; FeSO_4_ · 7H_2_O, 707.0; KCl, 1192.0; MgSO_4_ · 7H_2_O, 4317.0; MnSO_4_ · 4H_2_O, 31.0; KI, 5.3; NaCl, 4934.0; Na_2_SeO_3_·H_2_O, 3.4; ZnSO_4_ · 7H_2_O, 177.0; Ca(H_2_PO_4_)_2_·H_2_O, 12,457.0; KH_2_PO_4_, 9930.0.

^2^Vitamin premix supplied the following (IU or mg kg^−1^ diet): retinyl palmitate, 6500.0 IU; cholecalciferol, 2400.0 IU; *α*-tocopherol, 50.0 mg; menadione, 44.0 mg; thiamine HCl, 12.0 mg; riboflavin, 25.0 mg; D-calcium pantothenate, 20.0 mg; mesoinositol, 200.0 mg; D-biotin, 0.5 mg; folic acid, 1.5 mg; ascorbic acid, 100.0 mg; niacin, 75.0 mg; cyanocobalamin, 0.01 mg.

**Table 2:** Growth performance and feed utilization of coho salmon fed the diets with different pyridoxine levels for 10 weeks (means ± SE, *n* = 3).

Dietary pyridoxine levels (mg/kg)								*P* value
	0.32	1.25	2.56	4.08	8.24	16.02	32.32	
SR (%)	96.67 ± 3.33	96.67 ± 3.33	100.00 ± 0.00	96.67 ± 3.33	100.00 ± 0.00	100.00 ± 0.00	96.67 ± 3.33	0.879
IBW (g)	180.2 ± 0.13	181.0 ± 0.21	180.5 ± 0.23	180.4 ± 0.34	180.8 ± 0.23	180.2 ± 0.20	180.9 ± 0.41	0.985
FBW (g)	483.83 ± 3.31^a^	494.57 ± 3.28^a^	509.92 ± 3.22^b^	517.10 ± 1.80^b^	526.93 ± 3.68^c^	508.92 ± 0.67^b^	493.78 ± 0.94^a^	0.012
SGR (%/day)	1.72 ± 0.03^a^	1.75 ± 0.12^a^	1.81 ± 0.01^b^	1.82 ± 0.02^b^	1.87 ± 0.01 ^c^	1.80 ± 0.01^b^	1.75 ± 0.01^a^	0.011
CF	1.71 ± 0.03	1.79 ± 0.02	1.80 ± 0.02	1.83 ± 0.01	1.79 ± 0.03	1.77 ± 0.05	1.76 ± 0.03	0.542
HSI	0.76 ± 0.09	0.62 ± 0.06	0.59 ± 0.12	0.71 ± 0.12	0.58 ± 0.10	0.61 ± 0.09	0.59 ± 0.07	0.523
VSI	4.74 ± 0.40	4.31 ± 0.24	3.58 ± 0.46	3.57 ± 0.55	4.12 ± 0.54	4.11 ± 0.35	4.14 ± 0.08	0.632
FCR	1.72 ± 0.01^c^	1.68 ± 0.02^c^	1.60 ± 0.02^b^	1.58 ± 0.02^b^	1.51 ± 0.02^a^	1.61 ± 0.01^b^	1.68 ± 0.02^c^	0.013

*Note*: Means in the same raw with different superscript letters are significantly different (*P* < 0.05).

Abbreviations: CF, condition factor; FBW, final body weight; FCR, feed conversion ratio; HSI, hepatosomatic index; IBW, initial body weight; SR, survival rate; VSI, visceral somatic index.

**Table 3:** Muscle proximate composition of coho salmon fed the diets with different pyridoxine levels for 10 weeks (means ± SE, *n* = 3).

Dietary pyridoxine levels (mg/kg)	Moisture (%)	Crude protein (%)	Crude lipid (%)	Ash (%)
0.32	70.83 ± 0.52	13.12 ± 0.13	4.84 ± 0.01	3.55 ± 0.05
1.25	70.76 ± 0.07	13.10 ± 0.33	4.84 ± 0.02	3.21 ± 0.07
2.56	70.84 ± 0.65	13.11 ± 0.15	4.83 ± 0.03	3.36 ± 0.06
4.08	70.05 ± 0.13	13.27 ± 0.07	4.84 ± 0.04	3.72 ± 0.22
8.24	70.25 ± 0.08	13.09 ± 0.19	4.82 ± 0.03	3.65 ± 0.05
16.02	70.67 ± 0.07	13.12 ± 0.10	4.84 ± 0.04	3.63 ± 0.13
32.32	70.33 ± 0.09	13.15 ± 0.13	4.85 ± 0.01	3.45 ± 0.35
*P* value	0.546	0.741	0.321	0.258

*Note*: Non-significant (*P*  >  0.05).

**Table 4:** Effects of dietary pyridoxine levels on antioxidant enzymes and MDA content in the serum of coho salmon (means ± SE, *n* = 3).

Pyridoxine levels (mg/kg)	CAT (U/mL)	SOD (U/mL)	MDA (nmol/mL)
0.32	43.12 ± 2.13^a^	456.85 ± 5.36^a^	7.66 ± 0.06^b^
1.25	45.46 ± 0.07^a^	458.44 ± 4.92^a^	7.57 ± 0.06^b^
2.56	46.42 ± 1.43^a^	461.00 ± 2.28^a^	7.38 ± 0.02^a^
4.08	48.00 ± 0.30^b^	467.33 ± 3.30^b^	7.34 ± 0.03^a^
8.24	59.63 ± 0.40^c^	488.40 ± 4.20^c^	7.28 ± 0.03^a^
16.02	46.02 ± 1.45^a^	458.09 ± 3.23^a^	7.39 ± 0.01^ab^
32.32	46.82 ± 1.99^a^	454.03 ± 3.88^a^	7.44 ± 0.01^ab^
*P* value	0.01	<0.01	0.01

*Note*: Means in the same raw with different superscript letters are significantly different (*P* < 0.05).

Abbreviations: CAT, catalase; MDA, malondialdehyde; SOD, superoxide dismutase.

## Data Availability

The data will be made available on request.
